# Human breast milk isolated lactic acid bacteria: antimicrobial and immunomodulatory activity on the *Galleria mellonella* burn wound model

**DOI:** 10.3389/fcimb.2024.1428525

**Published:** 2024-09-06

**Authors:** Antonio Guarnieri, Noemi Venditti, Marco Alfio Cutuli, Natasha Brancazio, Giovanna Salvatore, Irene Magnifico, Laura Pietrangelo, Marilina Falcone, Franca Vergalito, Daria Nicolosi, Franco Scarsella, Sergio Davinelli, Giovanni Scapagnini, Giulio Petronio Petronio, Roberto Di Marco

**Affiliations:** ^1^ Università degli Studi del Molise Department of Medicina e Scienze della Salute “V. Tiberio”, Campobasso, Italy; ^2^ Unità Operativa (UO) Laboratorio Analisi, Responsible Research Hospital, Campobasso, Italy; ^3^ Università degli Studi del Molise Department of Agricultural, Environmental and Food Sciences, Campobasso, Italy; ^4^ Università degli Studi di Catania Department of Drug and Health Sciences, Catania, Italy; ^5^ ASReM-Azienda Sanitaria Regionale del Molise, Campobasso, Italy

**Keywords:** host-pathogen interaction, antimicrobial peptides, *Pseudomonas aeruginosa*, *Galleria mellonella*, burn wound infection model, lactic acid bacteria, immunomodulatory activity, burn infection prevention

## Abstract

**Introduction:**

Managing burn injuries is a challenge in healthcare. Due to the alarming increase in antibiotic resistance, new prophylactic and therapeutic strategies are being sought. This study aimed to evaluate the potential of live Lactic Acid Bacteria for managing burn infections, using *Galleria mellonella larvae* as an alternative preclinical animal model and comparing the outcomes with a common antibiotic.

**Methods:**

The antimicrobial activity of LAB isolated from human breast milk was assessed *in vitro* against *Pseudomonas aeruginosa* ATCC 27853. Additionally, the immunomodulatory effects of LAB were evaluated *in vivo* using the *G. mellonella* burn wound infection model.

**Results and discussion:**

*In vitro* results demonstrated the antimicrobial activity of Lactic Acid Bacteria against *P. aeruginosa*. *In vivo* results show that their prophylactic treatment improves, statistically significant, larval survival and modulates the expression of immunity-related genes, Gallerimycin and Relish/NF-κB, strain-dependently. These findings lay the foundation and suggest a promising alternative for burn wound prevention and management, reducing the risk of antibiotic resistance, enhancing immune modulation, and validating the potential *G. mellonella* as a skin burn wound model.

## Graphical Abstract

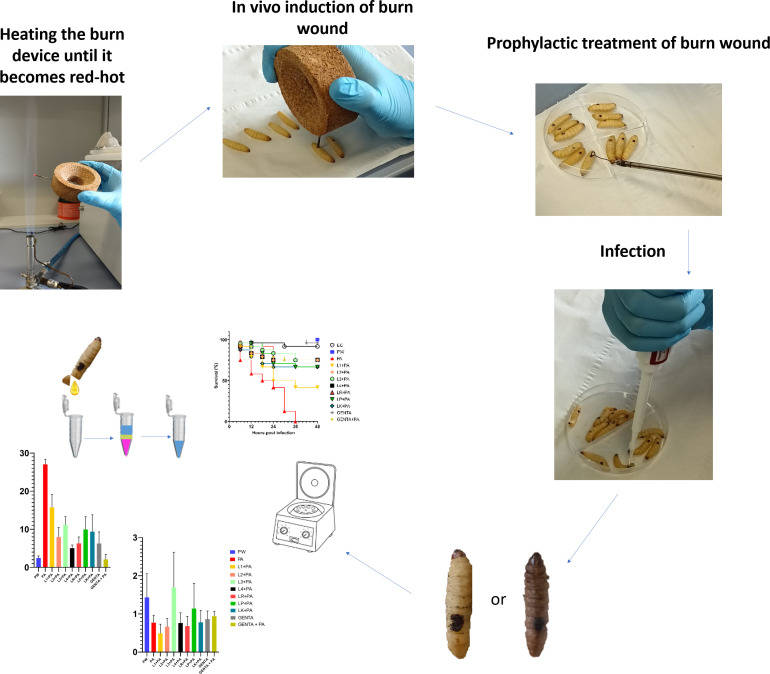

## 1 Introduction

Burn wounds represent a critical challenge in healthcare due to their potential to compromise skin, the body's first line of defense, thereby exposing individuals to the risk of infection and fluid imbalance. The burden of thermal injury worldwide, with more than 250,000 deaths reported each year, mainly affects populations in developing countries with limited access to adequate medical care (Rybarczyk et al., 2016). Often, internal injuries are stiffness in burns associated with extensive body surface area, and mortality reaches critical levels in severely burned patients (Forbinake et al., 2020). Despite efforts to maintain sterility, burn wounds are highly susceptible to microbial colonization and subsequent infection, particularly within the first week post-injury (Ladhani et al., 2021). The wound provides an ideal environment for the proliferation of microorganisms such as Gram-negative bacteria. The standard treatment for burn wounds includes wound care management, pain control, and the use of antibiotics to prevent or treat infections. Debridement, which involves the removal of dead tissue, is a critical step in preventing infection and promoting healing (Jeschke et al., 2020). Burn wounds often requiring aggressive antibiotic therapy to reduce the risk of systemic infection and sepsis. Traditionally, aminoglycosides, such as gentamicin, have been a cornerstone of antibiotic therapy in burn patients, exhibiting efficacy against a broad spectrum of bacteria, including opportunistic pathogens like *Pseudomonas. aeruginosa* (Corcione et al., 2021). However, the emergence of antibiotic resistance presents significant obstacles to successfully treating burn wound infections, necessitating alternative therapeutic strategies. These include the use of antimicrobial dressings, impregnated with silver or honey, which have shown efficacy in reducing bacterial load and promoting wound (Leaper et al., 2011). Other innovative approaches include the use of negative pressure wound therapy, which applies controlled suction to the wound area, thereby enhancing tissue perfusion and reducing edema (Huang et al., 2014; Agarwal et al., 2019; Norman et al., 2022). Furthermore, Lactic Acid Bacteria (LAB), particularly species within the *genus Lactobacillus*, have garnered attention for their potential antimicrobial and immunomodulatory properties (Argenta et al., 2016). LABs have demonstrated the ability to inhibit the growth of pathogenic bacteria, including *P. aeruginosa*, while simultaneously promoting wound healing processes (Andrejko et al., 2021). LABs are a promising option for future complementary therapy in burn wound prevention and management thanks to their diverse mechanisms, including competitive exclusion, generation of antimicrobial substances, and modulation of host immune responses. However, evaluating antimicrobial agents and immunomodulators in burn wound research is inherently challenging due to the complex nature of these injuries. *In vitro* models lack the complexity of burn wounds, while larger mammalian models present practical limitations such as high costs, ethical concerns, and logistical constraints (Hao and Nourbakhsh, 2021). The use of bacteria in burn injury and burn wound healing has been investigated by various groups in multiple *in vivo* animal studies with promising results (Maitz et al., 2023). In this context, *Galleria mellonella* offers a valuable tool for studying burn wound infections. Firstly, it avoids the ethical issues associated with vertebrate animal models. Additionally, it is an economical model that allows for high-throughput experimentation, facilitating the study of various treatments and the rapid and efficient screening of antimicrobial compounds. Also, the *G. mellonella* model can differentiate between high and low pathogenicity bacterial strains, making it useful for virulence studies, for testing the effectiveness of treatments against specific pathogens and it can be used to test *in vivo* bacterial antagonist activity. In recent years, many studies have shown that *G. mellonella* possesses an innate immune system, consisting of hemocytes, with the ability to produce antimicrobial peptides (AMP) and perform phagocytosis. It acts similarly to human innate immunity and shares many characteristics with mammals, including cellular and humoral defense mechanisms. The hemolymph of *G. mellonella larvae* is nearly germ-free, which significantly reduces the concern about internal contamination that might interfere with *in vivo* infection model studies and gene expression analysis (Kavanagh and Reeves, 2004; Venditti et al., 2021). Lastly, burns created on the surface of the *G. mellonella larvae* can replicate many aspects of human burns, including the progression of infection (Tsai et al., 2016; Lange et al., 2018; Maslova et al., 2020, 2021, 2023). Because of these characteristics, *G. mellonella* is a suitable alternative model to study the effectiveness of live LABs in prevention and control of burn wound infections. In fact, using this brand-new wound model, the purpose of this study is to assess the antibacterial and immunomodulatory activities of LABs isolated from human breast milk in the context of preventing burn wound infections and to investigate the regulation and modulation process of the *G. mellonella* immune system. This knowledge could help design more effective clinical approaches for the prophylactic treatment of human burn injuries in future (Maslova et al., 2020, 2021, 2023).

## 2 Materials and methods

A schematic study design representation is shown in **Supplementary Figure 1**.

### 2.1 Chemicals and reagents

De Man, Rogosa, and Sharpe (MRS) medium was purchased from Liofilchem and prepared according to manufacturer instructions (62g/L, pH 6.2).

Muller-Hinton (MH) was purchased from Liofilchem and prepared according to manufacturer instructions (21g/L, pH 7.3).

TRIzol Reagent User Guide (Invitrogen Waltham, MA, USA).

Gentamicin topical formulation produced by MSD Italia s.r.l.

Sodium Chloride (NaCl M=58.44g/mol) was purchased from PanReac AppliChem.

cDNA reverse transcription kit (Applied Biosystems™) with RNAse inhibitor.

qRT-PCR was performed using PowerUp™ SYBR™ Green Master Mix (Applied Biosystems).

Water, sterile, and molecular biology grade (DEPC treated, nuclease, and protease free) was purchased from HiMedia.

### 2.2 Bacterial strains

*P. aeruginosa* ATCC 27853 (PA) stored at -80°C with 20% glycerol.

#### 2.2.1 No-commercial LABs

*Leuconostoc citreum* DSM 34870 (L1), *Limosilactobacillus fermentum* DSM 34871 (L2), *Limosilactobacillus fermentum* DSM 34872 (L3) and *Limosilactobacillus fermentum* DSM 34873 (L4) stored at -80°C with 20% glycerol.

#### 2.2.2 Commercial LABs

*Lacticaseibacillus rhamnosus* ATCC 53103 (LR), *Lentilactobacillus kefiri* DSM 32079 (LK), *Lactoplantibacillus plantarum* ATCC 14917 (LP) stored at -80°C with 20% glycerol.

### 2.3 Bacterial preparations

LAB strains were inoculated into 15ml centrifuge tubes and Petri dishes containing MRS media. The cultures were grown anaerobically with the Anerogen (Thermo ScientificDiagnostic B.V. Landsmeer The Netherlands) anaerobic gas-generating sachet for 48 hours at 37°C (Linares-Morales et al., 2022).

*P. aeruginosa* strain was plated for growth on MH and incubated for 24 hours at 37°C (Sharma et al., 2020).

### 2.4 Antimicrobial susceptibility testing

The antibiotic susceptibility of *P. aeruginosa* ATCC 27583 was tested by Minimum Inhibitory Concentration (MIC) method as previously described by Blandino et al. and Petronio Petronio et al. with some modifications (Blandino et al., 2016; Petronio Petronio et al., 2020). Media, *inoculum* preparation, antibiotic dilution, and incubation conditions were chosen according to Clinical and Laboratory Standards Institute (CLSI, 2020). Gentamicin with a dilution range of 64-0.125µg/ml was used as test antibiotic. Also, the antibiotic susceptibility of *P. aeruginosa* ATCC 27583 was tested by the Disk Diffusion (DD) method as reported on the CLSI M100 (32nd Edition) guidelines. Gentamicin 10μg disk was used (Sami Awayid and Qassim Mohammad, 2022; Aggarwal et al., 2024).

### 2.5 Antimicrobial overlay assay

**Figure 1:**
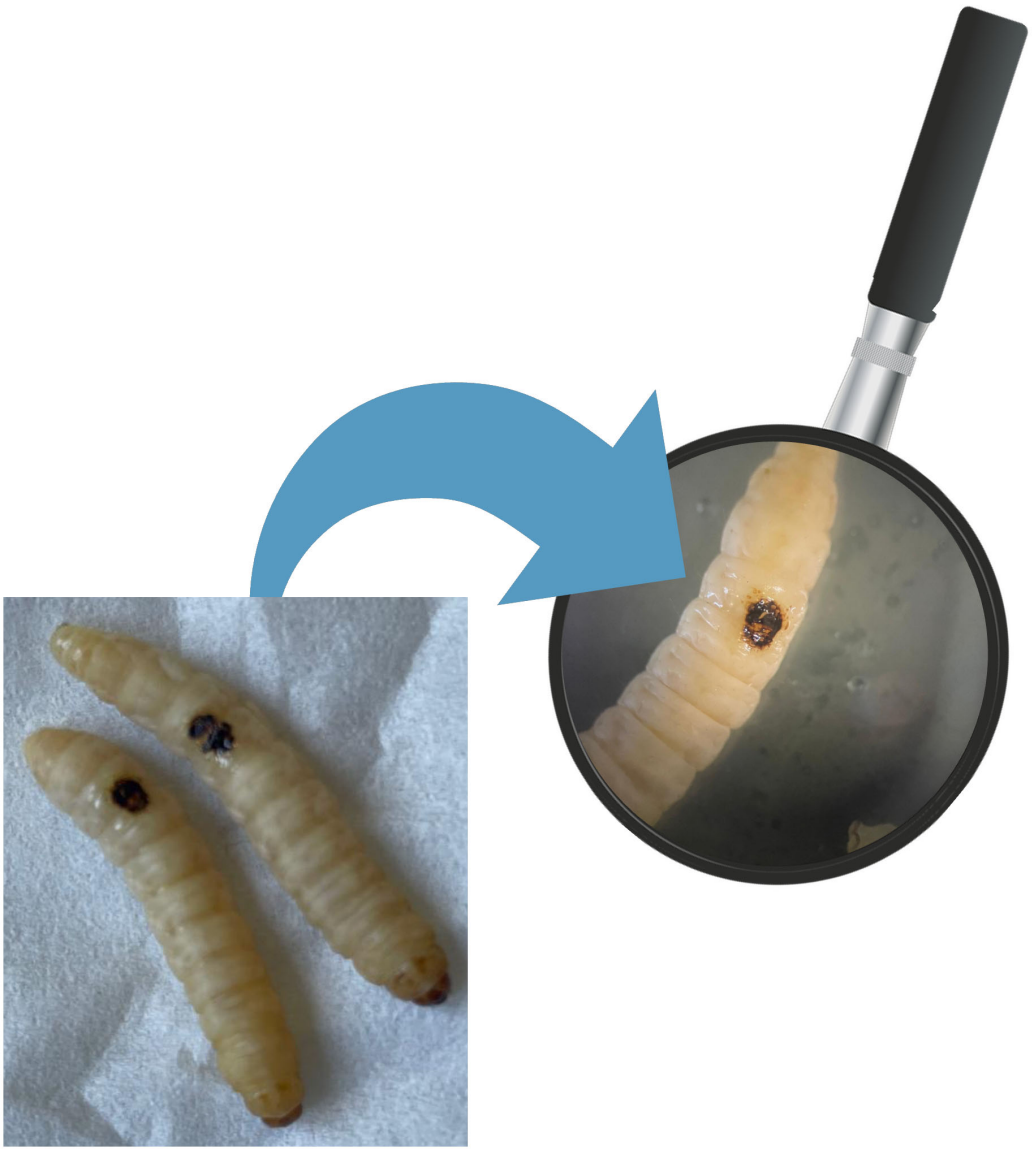
Burn wound on *G. mellonella* larvae.

The antimicrobial overlay assay was performed referring to the Hossain et al. (Hossain et al., 2022) method with some modifications. Briefly, a predefined volume of MRS was poured into each 90 mm Petri dish to prepare the basal agar layer. After agarization, a 10µl aliquot of 0.5 McFarland with a final *inoculum* concentration of 1 × 10^6^ CFU (Perkin Elmer Wallac Victor 3 1420 Multilabe, OD600) of each LAB strain was spotted in the center of the plate and, after a few minutes, incubated in anaerobic condition overnight at 37°C. The day after, an appropriate volume of the 0.5 McFarland suspensions of *P. aeruginosa* ATCC 27583 was added to the MH soft agar to seed it with a final *inoculum* concentration of 1 × 10^6^ CFU/ml. Then, the appropriate volume of soft agar was overlaid to the previously prepared base layer and after agarization, plates were re-incubated overnight at 37°C (Maricic and Dawid, 2014; Hockett and Baltrus, 2017; Riera et al., 2017). After incubation, all inhibition zones were measured by eye using a ruler to the nearest millimeter (**Figure 1**). The inhibition halos mean diameters were calculated from 3 replicates of 3 independent experiments.

### 2.6 *G. mellonella larvae* acquisition and preparation

The *larvae* were acquired as proposed by our previous work by Venditti et al. (Venditti et al., 2021). *G. mellonella larvae* were obtained from SA.GI.P. s.a.s. (Ravenna, Italy), kept at 15°C in darkness until use and used for experiments after 2 days of acclimatization at 35°C. The *larvae* chosen for the experiment weigh between 280-300mg and are in the final larval stage.

### 2.7 *In vivo* induction of burn wound on *G. mellonella* larvae

**Figure 2:**
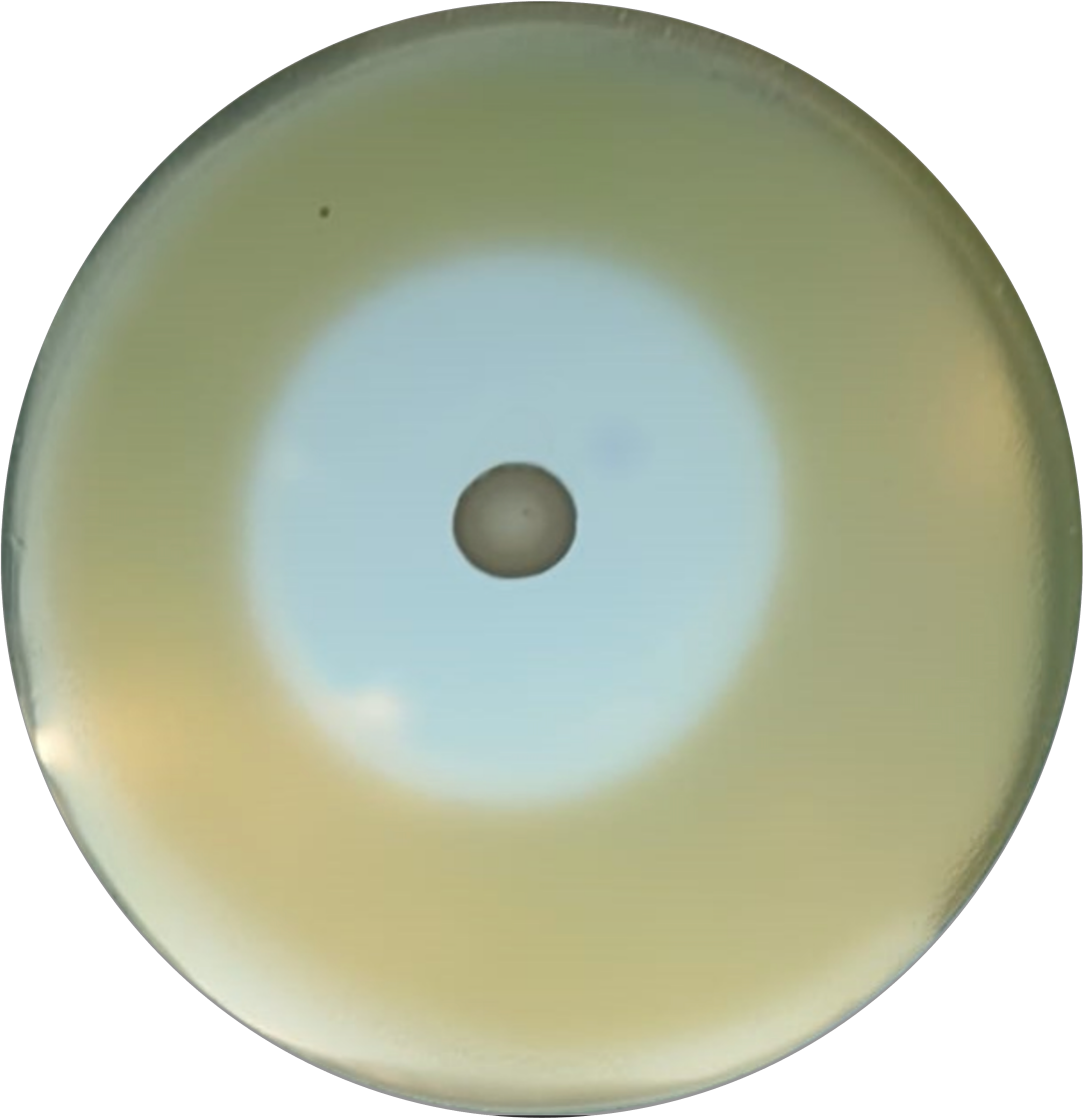
Zone of inhibition example by antimicrobial overlay assay.

The induction of *in vivo* burn wounds was conducted as described by Maslova et al (Maslova et al., 2020, 2023). Briefly, 70% ethanol was used to disinfect the surface of *G. mellonella larvae*, ensuring coverage of the entire larval body. Petri dishes were uncovered in a sterile environment to facilitate ethanol evaporation post-disinfection. Swabs of the disinfected *larvae* were plated on CLED media to verify the disinfection procedure. The *larvae* were positioned on their ventral side to expose the back segment and immobilized by securing the head and thorax segments. A burn instrument, a steel nail embedded in cork with a head size of 2mm², was heated in the central flame of a Bunsen burner until reaching a red/white-hot state and then applied to the middle segment of the *G. mellonella larvae*'s back for 4 seconds until a color variation (dark brown-black) in the cuticle was observed (**Figure 2**). This method ensures consistent burn wounds across specimens. *Larvae* displaying significant hemolymph loss or protruding fat body post-procedure were discarded from experimental setting and were promptly euthanized by exposure to temperatures of -20°C for at least 20 minutes to alleviate suffering. After all, the *larvae* were incubated at 35°C for 48 h (Maslova et al., 2020, 2023). Every experiment was done in triplicate and using an additional group of *larvae* naïve called "environmental control" (EC) verify the absence of interferences.

### 2.8 *Inoculum* preparation

*P. aeruginosa* ATCC 27583 *inoculum* was made as follow. Overnight culture grown in MH broth was pelleted and suspended in saline solution water up to an optical density of 0.1 at 600nm OD (Perkin Elmer Wallac Victor 3 1420 Multilabel) corresponding to 1.3 ± 0.2X10^8^ CFU/ml (Alghoribi et al., 2014).

### 2.9 Prophylactic treatment of burn wound using live LABs

Following burn induction, a sterile 10µl-loop transferred a colony of LAB strains from the MRS agar plates to the wound sites. After a 10-minute, 10µl of *P. aeruginosa inoculum* was pipetted onto the treated wound. The control groups received no treatment post-burn induction unless 10µl of saline solution (PW). The *larvae* were incubated at 35°C for 48 h. Mortality was recorded at 0, 6, 12, 18, 24, 36, 42, and 48 hours and was detected when a complete larval stillness occurred, even with external stimulation. Every following experiment was done in triplicate and using an additional group of naïve *larvae* (EC).

### 2.10 Prophylactic treatment of burn wound using 0.1% topical gentamicin

After burn induction, gentamicin 0.1% topical formulation was applied to the central part of the wound using a sterile 10µl-loop. After that, 10µl of *P. aeruginosa inoculum* was pipetted onto the treated wound. GENTA control group received only gentamicin 0.1% topical formulation treatment post-burn induction. PW group received no treatment post-burn induction unless 10µl of saline solution. The *larvae* were incubated at 35°C for 48 h. As previously detailed, mortality was recorded at 0, 6, 12, 18, 24, 36, 42, and 48 hours, every experiment was done in triplicate.

### 2.11 Burn wound infection

**Figure 3:**
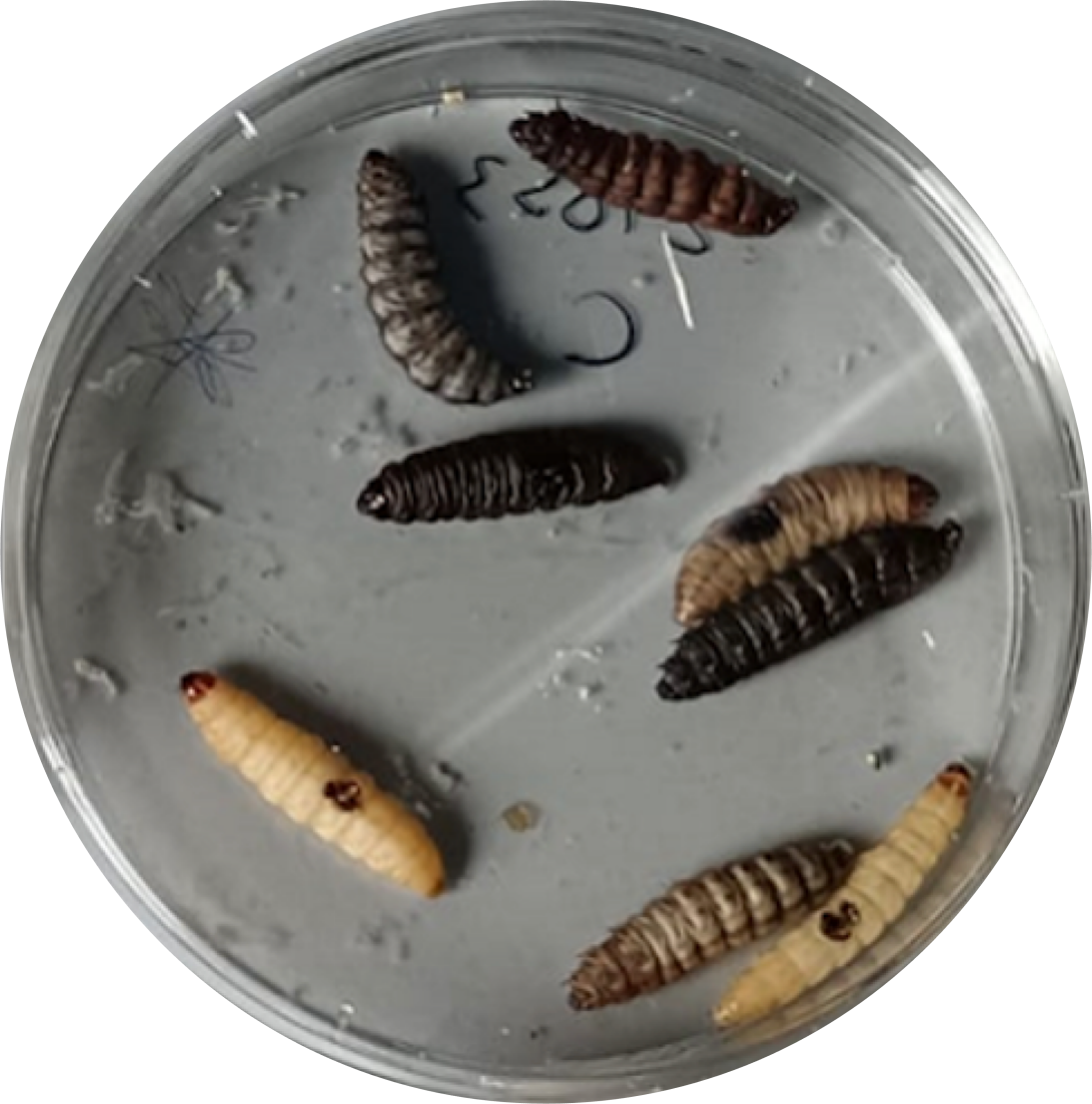
*G. mellonella larvae* treated with *P. aeruginosa* ATCC 2758.

10µl *P. aeruginosa inoculum* (final concentration: 1.3 ± 0.2X10^6^ CFU) was pipetted on burn wound *G. mellonella larvae* as described by Maslova et al. (Maslova et al., 2023). *Larvae* were incubated in 10cm plates at 35°C, and the number of dead *larvae* was scored 1 to 4 days after infection. The *larvae* was considered dead when it displayed no movement in response to touch (**Figure 3**). Twenty four *Larvae* were infected with *P. aeruginosa* only as infection control group (PA). Experiment was done in triplicate, eight *larvae* were tested in each replicate.

### 2.12 *G. mellonella* survival

24 *larvae* were distributed into 12 groups, as shown in **Table 1**.

**Table 1:** Names and numbers of *larvae* for each group.

GROUP NAME	EC	PW	PA	L1+PA	L2+PA	L3+PA	L4+PA	LR+PA	LP+PA	LK+PA	GENTA	GENTA+PA
N° *LARVAE*	24	24	24	24	24	24	24	24	24	24	24	24

EC = Naïve *larvae* control, PW = Burn wound *larvae* treated with Saline Solution, PA = Burn wound *larvae* infected with *P. aeruginosa* ATCC 27853, L1 + PA = Burn wound *larvae* pre-treated with L1 and infected with *P. aeruginosa* ATCC 27853, L2 + PA = Burn wound *larvae* pre-treated with L2 and infected with *P. aeruginosa* ATCC 27853, L3 + PA = Burn wound *larvae* pre-treated with L3 and infected with *P. aeruginosa* ATCC 27853, L4 + PA = Burn wound *larvae* pre-treated with L4 and infected with *P. aeruginosa* ATCC 27853, LR + PA = Burn wound *larvae* pre-treated with LR and infected with *P. aeruginosa* ATCC 27853, LP + PA = Burn wound *larvae* pre-treated with LP and infected with *P. aeruginosa* ATCC 27853, LK + PA = Burn wound *larvae* pre-treated with LK and infected with *P. aeruginosa* ATCC 27853, GENTA = Burn wound *larvae* treated with gentamicin topical formulation 0.1%, GENTA + PA = Burn wound *larvae* pre-treated with gentamicin topical formulation 0.1% and infected with *P. aeruginosa* ATCC 27853.

### 2.13 RNA extraction, cDNA amplification, and qRT-PCR gene expression

12 hours after both treatment, *G. mellonella larvae* were anesthetized at 4°C for 10 minutes. Subsequently, the last part of the *larvae* abdomen was cut off using a sterile surgical blade (Swann Morton Limited Sheffield S6 2BJ, England), and hemolymph was collected into 1.5 ml Eppendorf tubes while maintained on ice to prevent melanization (Moya-Andérico et al., 2020). Following the protocol outlined in the TRIzol Reagent User Guide (Invitrogen Waltham, MA, USA), RNA was extracted from the hemolymph of each larval group. A high-capacity cDNA reverse transcription kit (Applied Biosystems™) with RNAse inhibitor was used for cDNA amplification following the instructions outlined in the user guide. Quantitative real-time PCR (qRT-PCR) was performed using PowerUp™ SYBR™ Green Master Mix (Applied Biosystems) following the manufacturer's protocol utilizing Rotor-Gene Q (Qiagen). All the qRT-PCR reactions used specific gene primers for the antimicrobial peptide Gallerimycin (GAL) and transcription factor Relish/NF-kB (REL), as shown in **Table 2**. Results were normalized against the housekeeping gene Elongation factor 1-Alpha (EF1) level and shown as relative values compared with *larvae* naïve (EC group). Expression gene fold changes are expressed by the DeltaDeltaCT method (de Melo et al., 2013; Sarvari et al., 2020). The experiment was conducted using three biological replications for three technical replications.

**Table 2:** List of primers used (qRT-PCR).

PRIMER NAME	FORWARD	REVERSE	REFERENCES
Relish/NF-kB	TCCAAAAAGCACCCTACAATCG	GCACTTCGTAGCTCACATCTC	Sarvari et al., 2020
Elongation factor 1-Alpha	AACCTCCTTACAGTGAATCC	ATGTTATCTCCGTGCCAG	Dubovskiy et al., 2013
Gallerimycin	AACCATCACCGTCAAGCCA	TCGAAGACATTGACATCCATTGA	Sarvari et al., 2020

### 2.14 Statistical analysis

Survival estimates were calculated using the Kaplan-Meier method, with significance calculated from the log-rank approximation of the chi-square test (Wang et al., 2023). One-way analysis of variance (ANOVA) using Tukey's multiple-comparison test was applied to differentiate qRT-PCR data between groups (Liang et al., 2022). Correlation coefficients were calculated using non-parametric Spearman's rank correlation method. Statistical analyses were performed using SPSS Version 26.0. Armonk, NY: IBM Corp.

## 3 Results

### 3.1 Antimicrobial susceptibility testing

For *P. aeruginosa* ATCC 27853, the MIC of gentamicin is 2 µg/ml, and the inhibition halo diameter with 10 µg gentamicin is 17 mm. The results are shown in **Table 3**.

**Table 3:** MIC and DD of *P. aeruginosa* ATCC 27853 strain versus gentamicin.

STRAIN	MIC GENTAMICIN	INHIBITION HALO (10µg gentamicin)
*P. aeruginosa* ATCC 27853	2 µg/ml	17mm

### 3.2 Antimicrobial overlay assay

All LABs showed excellent antibacterial activity against PA, except for L2 (**Table 4**). LABs isolated from human breast milk (No-commercial LABs) inhibited the growth of PA to an equal or greater extent than commercial strains; in fact, L3 had the highest antimicrobial activity *in vitro* (45mm) followed by LK (43mm), LP and L4 in a tie (40mm).

**Table 4:** Results of the agar overlay assay against *P. aeruginosa* ATCC 27853.

SAMPLE	ZONE OF INHIBITION (mm)
*Leuconostoc citreum DSM 34870 (L1)*	*36 ± 2*
*Limosilactobacillus fermentum* DSM 34871 (L2)	28 ± 2
*Limosilactobacillus fermentum* DSM 34872 (L3)	45 ± 1
*Limosilactobacillus fermentum* DSM 34873 (L4)	40 ± 2
*Lacticaseibacillus rhamnosus* ATCC 53103 (LR)	33 ± 1
*Lentilactobacillus kefiri* DSM 32079 (LK)	43 ± 1
*Lactoplantibacillus plantarum* ATCC 14917 (LP)	40 ± 1

The dimensions of the observed inhibition halos represent the mean of the three replicates of three independent experiments.

### 3.3 Burn wound infection on *G. mellonella*

As previously described, 10µl *P. aeruginosa inoculum* was administered in *G. mellonella larvae* and the mortality rate after 24 hours was 87.50% (**Table 5**).

**Table 5:** *G. mellonella* burn wound infection.

Strain	OD600	CFU/ml	Mortality rate (24h)
**PA**	0.1	1.3 ± 0.2X10^8^	87.50%

The mortality percentage was calculated by summing thenumber of dead larvae across the three replicates and dividing this valueby the total number of larvae at 24h. PA, *P. aeruginosa* ATCC 27853.

### 3.4 LABs colonization improves *G. mellonella* survival

**Figure 4:**
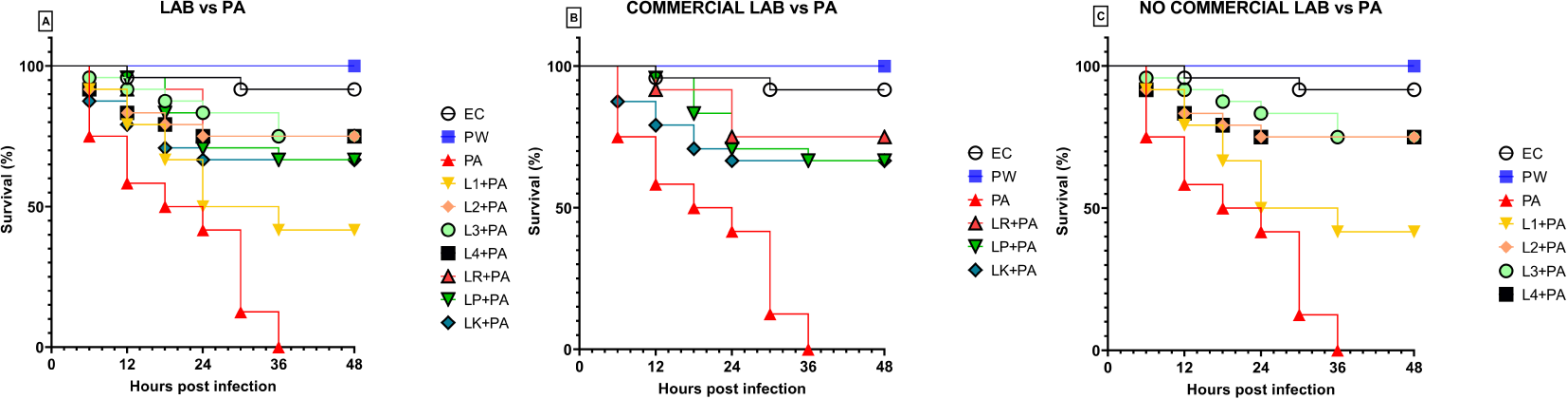
Survival curves of *in vivo* burn wounds pre-treated with all LABs **(A)**, commercial LABs **(B)**, and no commercial LABs **(C)** vs PA. EC = Naïve *larvae* control, PW = Burn wound *larvae* treated with Saline Solution, PA = Burn wound *larvae* infected with *P. aeruginosa* ATCC 27853, L1 + PA = Burn wound *larvae* pre-treated with L1 and infected with *P. aeruginosa* ATCC 27853, L2 + PA = Burn wound *larvae* pre-treated with L2 and infected with *P. aeruginosa* ATCC 27853, L3 + PA = Burn wound *larvae* pre-treated with L3 and infected with *P. aeruginosa* ATCC 27853, L4 + PA = Burn wound *larvae* pre-treated with L4 and infected with *P. aeruginosa* ATCC 27853, LR + PA = Burn wound *larvae* pre-treated with LR and infected with *P. aeruginosa* ATCC 27853, LP + PA = Burn wound *larvae* pre-treated with LP and infected with *P. aeruginosa* ATCC 27853, LK + PA = Burn wound *larvae* pre-treated with LK and infected with *P. aeruginosa* ATCC 27853.

Previously, the survival rate of burned *larvae* treated with LABs only was evaluated, and no mortality was found (data not shown). Thereafter, the protective activity of LABs against PA can be observed in **Figures 4A–C**. *Larvae* of the PA group all die at 36h after infection, whereas LABs pre-treated *larvae* survived up to 48h. At 20h, LR emerges as the strain with the best protective activity. At 48h after infection, L1 showed a minor protective activity, reducing mortality by 41.7%, followed by LK and LP, reducing mortality by 66.7%, and the remaining ones, L2, L3, L4, and LR, respectively, reducing mortality by 75%. Statistical analysis of L1, L2, L3, L4, LR, LP, and LK prophylactic treatment groups showed a significant reduction in mortality compared with the PA group (**Table 6**). These results suggest that each of the LABs demonstrated efficacy in *in vivo* antimicrobial activity. On the other hand, no significant differences were observed between L1, L2, L3, and L4 strains compared with LR, LP, and LK strains.

**Table 6:** Survival estimates were calculated using the Kaplan-Meier method, with significance from the log-rank approximation of the chi-square test.

	EC	PW	PA	L1+PA	L2+PA	L3+PA	L4+PA	LR+PA	LP+PA	LK+PA	GENTA	GENTA+PA
EC		0.1529	**≤0.0001**	**≤0.001**	0.1135	0.1281	0.1135	0.1262	**≤0.05**	**≤0.05**	0.5386	0.1252
PW	0.1529		**≤0.0001**	**≤0.0001**	**≤0.01**	**≤0.01**	**≤0.01**	**≤0.01**	**≤0.01**	**≤0.01**	0.3173	**≤0.01**
PA	**≤0.0001**	**≤0.0001**		**≤0.01**	**≤0.0001**	**≤0.0001**	**≤0.0001**	**≤0.0001**	**≤0.0001**	**≤0.0001**	**≤0.0001**	**≤0.0001**
L1+PA	**≤0.001**	**≤0.0001**	**≤0.01**		**≤0.05**	**≤0.05**	**≤0.05**	**≤0.05**	0.0675	0.1556	**≤0.0001**	**≤0.05**
L2+PA	0.1135	**≤0.01**	**≤0.0001**	**≤0.05**		0.9140	1.0000	0.8604	0.6763	0.5280	**≤0.05**	0.8789
L3+PA	0.1281	**≤0.01**	**≤0.0001**	**≤0.05**	0.9140		0.9140	0.9823	0.5430	0.4441	**≤0.05**	0.9949
L4+PA	0.1135	**≤0.01**	**≤0.0001**	**≤0.05**	1.0000	0.9140		0.8604	0.6763	0.5280	**≤0.05**	0.8789
LR+PA	0.1262	**≤0.01**	**≤0.0001**	**≤0.05**	0.8604	0.9823	0.8604		0.5195	0.3956	**≤0.05**	0.9837
LP+PA	**≤0.05**	**≤0.01**	**≤0.0001**	0.0675	0.6763	0.5430	0.6763	0.5195		0.7890	**≤0.01**	0.5407
LK+PA	**≤0.05**	**≤0.01**	**≤0.0001**	0.1556	0.5280	0.4441	0.5280	0.3956	0.7890		**≤0.01**	0.4116
GENTA	0.5386	0.3173	**≤0.0001**	**≤0.0001**	**≤0.05**	**≤0.05**	**≤0.05**	**≤0.05**	**≤0.01**	**≤0.01**		**≤0.05**
GENTA+PA	0.1252	**≤0.01**	**≤0.0001**	**≤0.05**	0.8789	0.9949	0.8789	0.9837	0.5407	0.4116	**≤0.05**	

Pairwise Comparisons are shown. p ≤ 0.05=*, p ≤ 0.01=**, p ≤ 0.001=***, p ≤ 0.0001=****.

### 3.5 LABs colonization matches the antibiotic activity of gentamicin topical formulation

**Figure 5:**
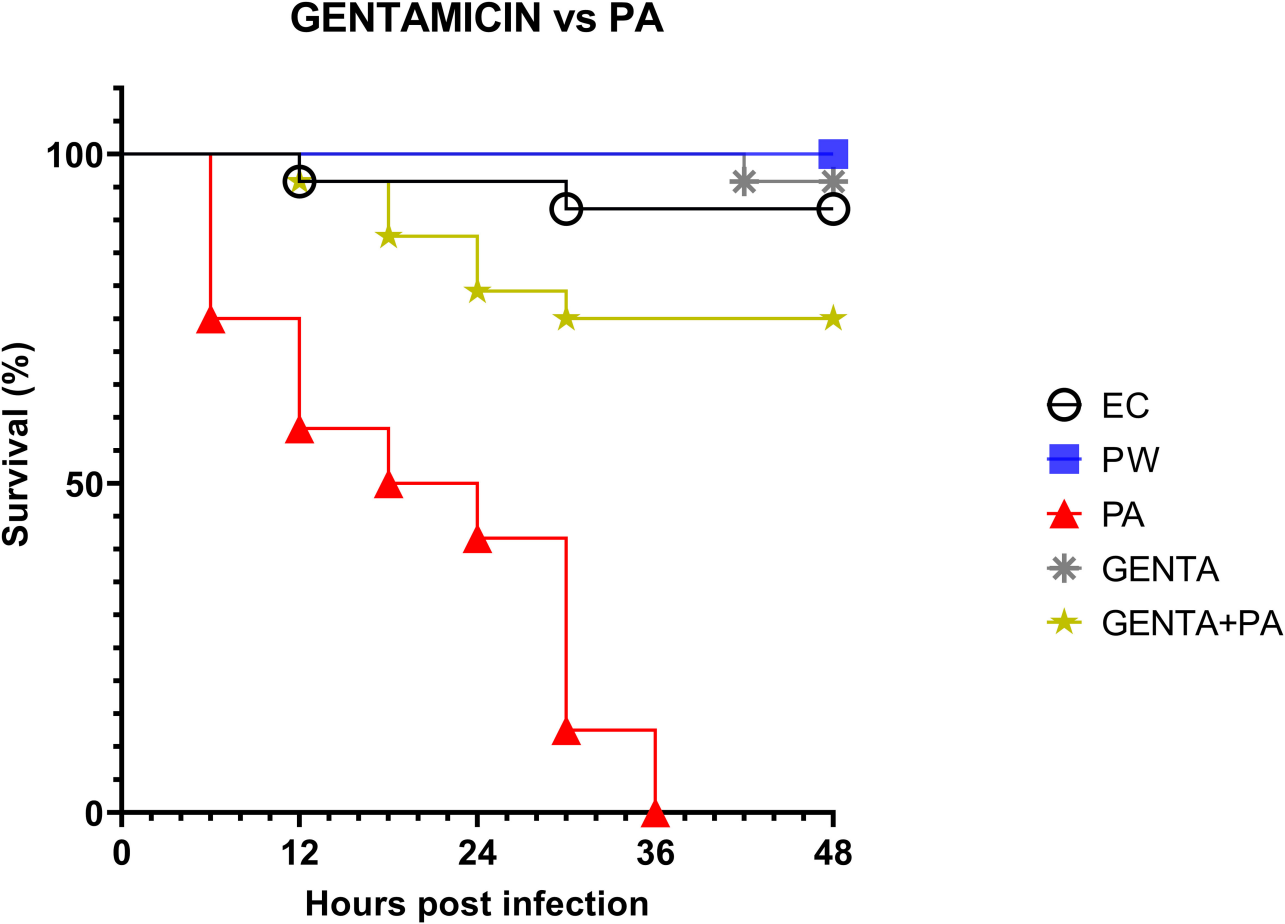
Survival curves of *in vivo* burn wound treatment with gentamicin topical formulation vs PA. EC = Naïve *larvae* control, PW = Burn wound *larvae* treated with Saline Solution, PA = Burn wound *larvae* infected with *P. aeruginosa* ATCC 27853, GENTA = Burn wound *larvae* treated with gentamicin topical formulation 0.1%, GENTA + PA = Burn wound *larvae* pre-treated with gentamicin topical formulation 0.1% and infected with *P. aeruginosa* ATCC 27853.

As described above, *larvae* of the PA group all die at 36h after infection. At 48 hours, *larvae* pre-treated with the topical formulation of gentamicin, compared to the group infected with PA, showed 75% reduced mortality. As shown in **Figure 5**, both LABs and gentamicin prophylactic treatments induced a significant and similar reduction in infections compared with controls (p ≤ 0.001) (**Figure 4**).

### 3.6 mRNA expression levels of Gallerimycin and Relish/NF-kB

**Figure 6:**
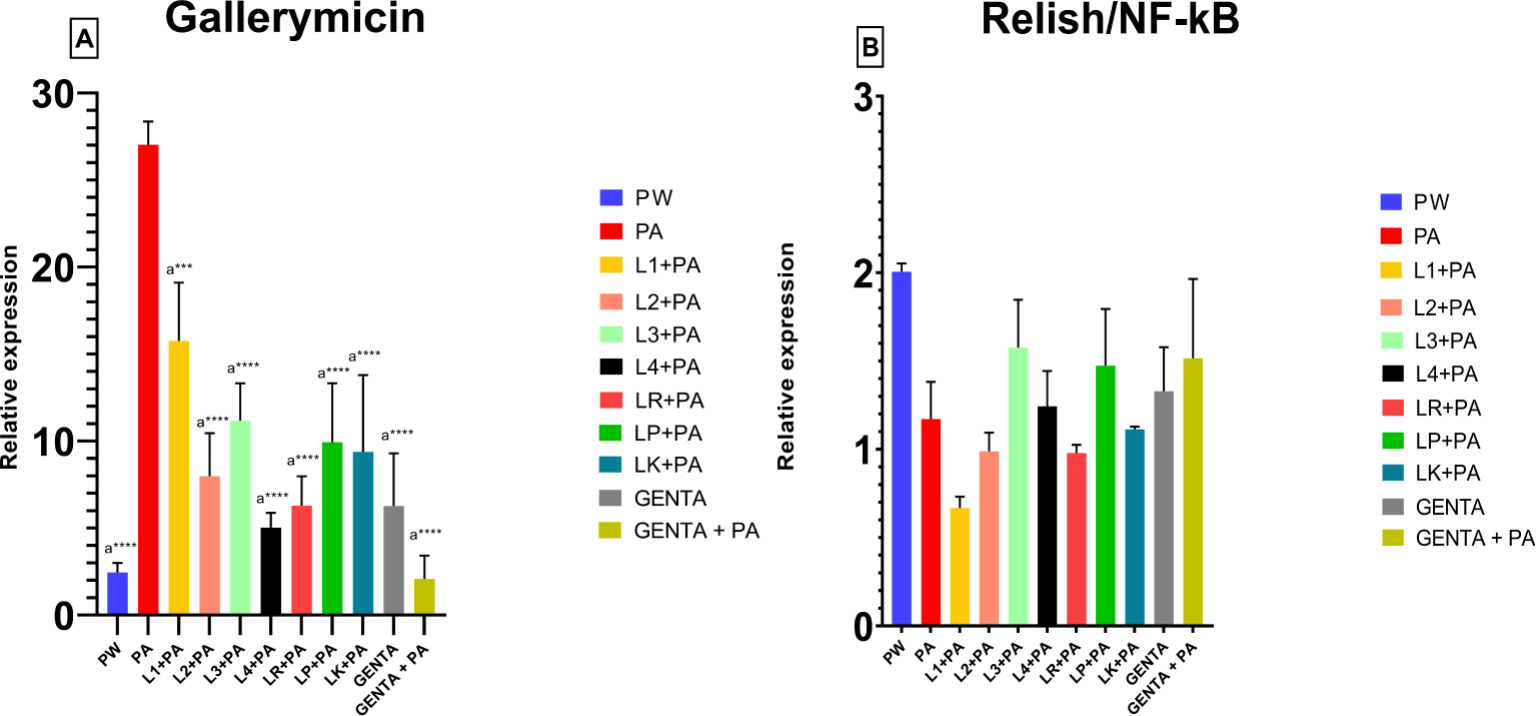
Gallerimycin **(A)** mRNA levels and Relish/NF-kB **(B)**. PW = Burn wound *larvae* treated with Saline Solution, PA = Burn wound *larvae* infected with *P. aeruginosa* ATCC 27853, L1 + PA = Burn wound *larvae* pre-treated with L1 and infected with *P. aeruginosa* ATCC 27853, L2 + PA = Burn wound *larvae* pre-treated with L2 and infected with *P. aeruginosa* ATCC 27853, L3 + PA = Burn wound *larvae* pre-treated with L3 and infected with *P. aeruginosa* ATCC 27853, L4 + PA = Burn wound *larvae* pre-treated with L4 and infected with *P. aeruginosa* ATCC 27853, LR + PA = Burn wound *larvae* pre-treated with LR and infected with *P. aeruginosa* ATCC 27853, LP + PA = Burn wound *larvae* pre-treated with LP and infected with *P. aeruginosa* ATCC 27853, LK + PA = Burn wound *larvae* pre-treated with LK and infected with *P. aeruginosa* ATCC 27853, GENTA = Burn wound *larvae* treated with gentamicin topical formulation 0.1%, GENTA + PA = Burn wound *larvae* pre-treated with gentamicin topical formulation 0.1% and infected with *P. aeruginosa* ATCC 27853. ***=p ≤ 0.001, ****=p ≤ 0.0001, "a" indicates statistical significance toward the PA group.

The mRNA levels of Gallerimycin and Relish/NF-κB of burn wound *larvae* are summarized in **Figures 6A, B** respectively. A highly significant difference in Gallerimycin mRNA levels was observed between the PA and PW control groups (p ≤ 0.0001), indicating a substantial impact of *P. aeruginosa* infection on gene expression compared to the control group (**Figure 6A**). Likewise, between PA control group and L1+PA group a significant difference (p ≤ 0.001) was recorded as well as for all other treatments (L2+PA, L3+PA, L4+PA, LR+PA, LP+PA, LK+PA, GENTA, GENTA+PA) p ≤ 0.0001 (**Figure 6A**). The mRNA levels of Relish/NF-κB did not show significant differences compared to control (**Figure 6B**). In addition, there was a statistically significant negative correlation among PA infected, L1 and L3 treated *larvae*. In contrast, L2 and L4 a statistically significant positive correlation was found. While, for the other treatment groups, no statistically significant correlations have been found (**Table 7**).

**Table 7:** Correlation coefficients were calculated using non-parametric Spearman's rank correlation method.

	PA REL	L1+PA REL	L2+PA REL	L3+PA REL	L4+PA REL
PA GAL	-1.000**				
L1+PA GAL		-1.000**			
L2+PA GAL			+1.000**		
L3+PA GAL				-1.000**	
L4+ PA GAL					+1.000**

p ≤ 0.01=**.

## 4 Discussion

Severe burns are very devastating forms of trauma that require immediate and specialized medical care. The immunosuppression state, triggered by the burn trauma, and the wound's local microenvironment are favorable for microbial colonization and proliferation. Of major concern among the bacterial etiopathological agents of infections is the opportunistic pathogen *P. aeruginosa*, Gram-negative, facultative anaerobic rods, non-fermentative, a-sporogenous, motile by polar flagellum (Fakhry and Aljanabi, 2024) causing severe delays in the healing of burn patients and/or leading to *exitus*, mainly due to multi-resistant strains (Azzopardi et al., 2014; Fournier et al., 2016; Gonzalez et al., 2016).

The work by Cutuli et al. extensively describes the key attributes of *G. mellonella* in microbiology, as well as its application as an *in vivo* model for the advancement of novel antibacterial strategies in 2019 (Cutuli et al., 2019). Moreover, Maslova et al, studying *G. mellonella larvae* and using them as burn model, recapitulate the hallmarks of burn trauma and infection seen in mammalian models (Maslova et al., 2020, 2023). Therefore, using this suitable model, our paper investigated the antimicrobial and immunomodulatory properties of live LABs derived from human breast milk compared to three commercial strains (*L. plantarum, L. kefiri*, and *L. rhamnosus*) in burn wound infection management (Köhler, 2015; Champion et al., 2016). *In vitro* antimicrobial activity performed by overlay assay unveiled the strong inhibition activity of tested LABs against *P. aeruginosa* ATCC 27853. The overlay assay is a fast-preliminary screening tool widely used in antimicrobial research. It is reliable, simple, inexpensive, and easy to interpret. This method makes it possible to observe and compare specific zones of inhibition that can be critical in identifying active compounds or interactions between different microorganisms (Maricic and Dawid, 2014; Hockett and Baltrus, 2017; Riera et al., 2017; Hossain et al., 2022). In our study, we used whole cells of human milk-derived LABs to closely mimic the natural conditions where live *Lactobacillus* interact with pathogens. The presence of whole and live cells is crucial for evaluating not just the antimicrobial compounds they produce but also the potential synergistic effects of probiotic cells in combating infections (Hernández et al., 2005; Salas-Jara et al., 2016). While the use of cell-free supernatants (CFS) can provide insights into the antimicrobial compounds secreted by the bacteria, our focus was to evaluate the comprehensive antimicrobial activity, including the direct interactions of live cells with the pathogens. Therefore, we used whole cells to capture this holistic effect. In addition, to ensure proper evaluation of antimicrobial activity during the overlay assay, the incubation period and growth conditions were set so that primary antimicrobial effects could be observed without significant influence from further cell proliferation. Notably, *L. fermentum* DSM 34872 (L3) exhibited remarkable inhibitory effects on *P. aeruginosa* growth, outperforming both the commercial strains tested and the DD test (10µg gentamicin disk); followed by *L. kefiri* DSM 32079 (LK), *L. fermentum* DSM 34873 (L4) and *L. plantarum* ATCC 14917 (LP) in a tie (**Table 4**). Our results agree with previous *in vitro* findings about LABs antimicrobial activity versus several bacterial pathogens, including *P. aeruginosa* (Azzopardi et al., 2014). LAB's ability to produce antimicrobial compounds and compete for niche colonization likely underlies their effectiveness in inhibiting bacterial growth (Varma et al., 2011). However, a direct comparison between overlay assay and DD method is not possible due to the different methodologies employed. The overlay assay involves inoculating the bacteria into a soft agar medium that is overlaid onto a pre-inoculated base agar, whereas the DD method involves applying antibiotic-impregnated disks onto a pre-inoculated agar surface. Despite these strong differences, the DD method can still provide valuable insights, especially when interpreting results obtained with gentamicin (Gaudreau et al., 2008; Hossain et al., 2022). Moreover, *in vitro* LABs antimicrobial activity was compared to the *in vivo* treated *larvae* survival rate. The results were consistent across all LABs tested, except for the L2 strain (**Figures 4A–C**). Survival rates were compared between the PW control group and the PA control group with all prophylactic conditions to determine the specific impact. The survival data indicated no mortality in the heat-treated control group (PW) (**Figures 4A–C**), confirming that observed deaths were due to *P. aerugionosa* infection rather than burn wound induction. Although L2 *in vitro* inhibition halo was the lowest, thus suggesting weak antimicrobial activity against *P. aeruginosa*. In contrast, L2 *in vivo* results showed the best activity along with L3 and L4, reducing *larvae* mortality rates by 75%. This conflicting result confirms that *in vitro* experimentation cannot always be replaced *in vivo* experiments (Lorian, 1988). Moreover, this study compared the efficacy of live LABs prophylactic treatment with gentamicin topical formulation prophylactic treatment, an aminoglycoside commonly employed antibiotic in burn wound management (Corcione et al., 2021). These findings align with prior literature, indicating that gentamicin outperforms all other antibacterial tested against *P. aeruginosa*, significantly reducing bacterial counts in burn-injured porcine tissue (Nuutila et al., 2020). Intriguingly, either LABs (especially L2, L3, L4, and *L. rhamnosus* ATCC 53103, namely LR) and gentamicin showcased the same protective effects against *P. aeruginosa* infection in the *G. mellonella* model (**Figures 4A–C**). Lastly, mRNA expression analysis on hemolymph revealed significant alterations in the expression levels of immune-related genes, particularly Gallerimycin, in response to *P. aeruginosa* infection and LABs prophylactic treatments (**Figure 6A**). Previous studies have demonstrated distinct immune responses in *G. mellonella* to different types of microorganisms. Their immune response to pathogenic bacteria like *P. aeruginosa* typically includes upregulation of specific AMP and increased hemocyte activity (Tsai et al., 2016). In contrast, non-pathogenic bacteria such as LAB often induce a milder immune response (Mastromarino et al., 2014). The Gallerimycin is a pivotal AMP in the innate immune response of *G. mellonella*. Structurally, Gallerimycin shares similarities with defensins found in other insects and even certain vertebrates, suggesting a conserved mechanism of action against pathogens across species (Tsai et al., 2016; Ménard et al., 2021). Reported findings in this paper confirm a substantial upregulation of Gallerimycin expression in *larvae* infected with *P. aeruginosa*, denoting the activation of the larval immune system in response to bacterial challenge (**Figure 6A**). Worth mentioning, LABs prophylactic treatment attenuated the upregulation of Gallerimycin, suggesting a modulation of the immune response towards a less inflammatory phenotype described in literature (Andrejko et al., 2021). Gallerimycin was known exclusively for fungi and not for gram positive bacteria or yeasts (Schuhmann et al., 2003) but several studies suggest that its expression is also induced by infection of Gram-negative bacteria (Bolouri Moghaddam et al., 2016; Andrejko et al., 2021). Relish/NF-κB, the second gene studied, is a critical transcription factor in the Immune deficiency (IMD) pathway of insects, analogous to the NF-κB pathway in vertebrates. The IMD pathway is an essential component of the innate immune system, responsible in the early stages of the immune response for defense against microbial infections, particularly those caused by Gram-negative bacteria, as outlined in numerous studies on insect models, including our prior research on *Tenebrio molitor* immunity (Sarvari et al., 2020; Petronio Petronio et al., 2022) and other on *Drosophila melanogaster* (Cammarata-Mouchtouris et al., 2022; Mahanta et al., 2023). qRT-PCR analysis showed mild mRNA expression changes at 12 hours of treatments. Specifically, hypo-expression was observed in all conditions except for the PW control group, L3 group, and LP group, which showed mild overexpression (**Figure 6B**). Spearman's correlation analysis performed beyond the Relish/NF-κB and Gallerimycin mRNA fold expression revealed a statistically significant correlation in a strains-dependent manner in no-commercial LABs (**Table 7**). Although this is a pioneering study correlating the expression of Relish/NF-κB and Gallerimycin in infected *G. mellonella larvae*, a possible explanation can be found in previous studies conducted in *Drosophila*. Indeed, the correlation found in human breast milk isolated LABs treated *larvae*, reveals a possible feedback mechanism exerted by the expression of Gallerimycin against Relish/NF-κB as already observed in *Drosophila* where Relish/NF-κB acts as a controller to avoid unnecessary overexpression of AMP during the acute phase of infection. Pan et al. (Pan et al., 2023) demonstrated how Relish/NF-κB can flexibly alternate its role from a positive regulator to an indirect negative regulator via directly activating miR-275 in balancing *Drosophila* immune responses. Furthermore, prolonged overexpression of Relish/NF-κB can reduce *Drosophila* lifespan (Badinloo et al., 2018) and/or cytotoxicity phenomena (Stączek et al., 2023). After all, this control mechanism is also preserved in more complex animal species, including humans. NF-κB is one of the most significant transcription factors that control inducible gene expression as cells attempt to restore homeostasis and must be subject to strict spatiotemporal control to ensure measured and context-specific cellular responses during infection (Prescott et al., 2021). All this corroborates the immunomodulatory capacity of live LABs.

In conclusion, our study demonstrated that prophylactic treatment with live LABs is effective against *P. aeruginosa* both *in vitro* and *in vivo*. Moreover, the activity of some strains is comparable to the prophylactic action of gentamicin. Additionally, LABs can modulate the host immune system, enhancing anti-pathogenic capabilities and reducing harmful inflammatory responses. This paper has significant innovative potential because it explores a new avenue for burn wound treatment. This approach, in addition to preventing possible opportunistic infections, could reduce reliance on traditional antibiotics, thereby helping to mitigate the global issue of antibiotic resistance. As mentioned, the mechanisms by which this occurs aren't precise yet but include several possibilities. LABs may physically occupy space in the burned tissues that would then play host to pathogenic bacteria, blocking them from a residence in the injured tissue. Since LABs have already exhibited inhibitory activity *in vitro* against *P. aeruginosa* (Shokri et al., 2018), there is more to it than mechanical action. Their presence probably induces acidification of the local tissue environment, creating conditions unfavorable for the growth of pathogens (Argenta et al., 2016). Additionally, LABs produce substances that hinder the physiological processes of *P. aeruginosa*, for example, fermenticin from *fermentum* strains (Kaur et al., 2013), plantaricin from *plantarum* strain (Righetto et al., 2023), and a famous example is reuterin from *L. reuteri*, molecules with remarkable antimicrobial activity (Asare et al., 2020). Moreover, LABs may modulate the host immune system, enhancing their anti-pathogenic capabilities and dampening detrimental inflammatory reactions (Thoda and Touraki, 2023). *G. mellonella* model also represents an advancement in preclinical research methodologies. As previously discussed, the immunomodulatory potential of LABs prophylactic treatments in *G. mellonella* model is attributed to the modulation of Gallerimycin and Relish/NF-κB expression, the strain-specific effects of LABs. Relish/NF-κB, influenced by LABs, can flexibly alternate its role from a positive regulator to an indirect negative regulator thanks to a feedback mechanism regulating expressions of AMP. Laying the foundation on this new line of research, recent studies used LABs treatment on *ex-vivo* human skin models (Li et al., 2023) and used *L. plantarum* on *in vivo* burn wounds, with remarkable results (Peral et al., 2009).

Despite the premises, several limitations should be acknowledged. The *G. mellonella larvae*, as the other *in vivo* alternative models as emphasized in our earlier investigation conducted by Cutuli et al. in 2021, employing the snail model *Limacus flavus* for *in vivo* assessment of mucosal irritation (Cutuli et al., 2021). Indeed, alternative models may not fully recapitulate the complexity of human burn wounds. In fact, *G. mellonella* does not possess the capability to fully heal burn wounds as complex organisms do. Due to their biology, *larvae* typically enter pupation with the burn wound still present, and the lesion does not regenerate (Maslova et al., 2020; Pereira et al., 2020). This characteristic limits the extent to which we can assess complete wound healing in this model. As such, while the *G. mellonella* model is valuable for studying the effects of antimicrobial treatments and general health impacts, it has inherent limitations in evaluating full tissue regeneration and wound closure (Dai et al., 2011; Kazek et al., 2019; Maslova et al., 2020; Pereira et al., 2020; Serrano et al., 2023). However, future studies should validate their efficacy in more clinical experiments, for example, comparing results in validated *in vivo* models, such as murine models, to affirm the real translational potential (Cutuli et al., 2019). Continued research is needed to understand how LABs influence both pathogens and hosts. Additional clinical trials exploring their application in burn and other wound scenarios will help clarify the expanding role of prophylactic live LABs treatment and their immunomodulatory action. This will enhance our understanding of their potential to prevent infections and promote healing, providing a viable alternative to traditional antibiotics.

## Data Availability

The datasets presented in this study can be found in online repositories. “Figshare” data repository at https://DOI.org/10.6084/m9.figshare.25594491.
