# Supplementary material for: Human breast milk isolated lactic acid bacteria: antimicrobial and immunomodulatory activity on the Galleria mellonella burn wound model
Source: Front Cell Infect Microbiol. 2024 Sep 6;14:1428525. doi: 10.3389/fcimb.2024.1428525 (PMC11412949; doi:10.3389/fcimb.2024.1428525)

***Supplementary Material***

**Supplementary Figure 1.** Schematic study design representation. *in vitro* experiments; *in vivo* experiments experimental groups disinfection procedure; experimental groups burning procedure; Environmental control (EC): *naïve larvae* were not handled to verify the absence of environmental interference; Physiological water (PW): after disinfection *larvae* were burned and treated with sterilized saline solution to exclude possible exogenous sources of infection; PA: following disinfection and burning procedures *larvae* were infected with *P. aeruginosa* as the infection control group; GENTA: following disinfection and burning procedures *larvae* were treated with gentamicin to assess toxicity.


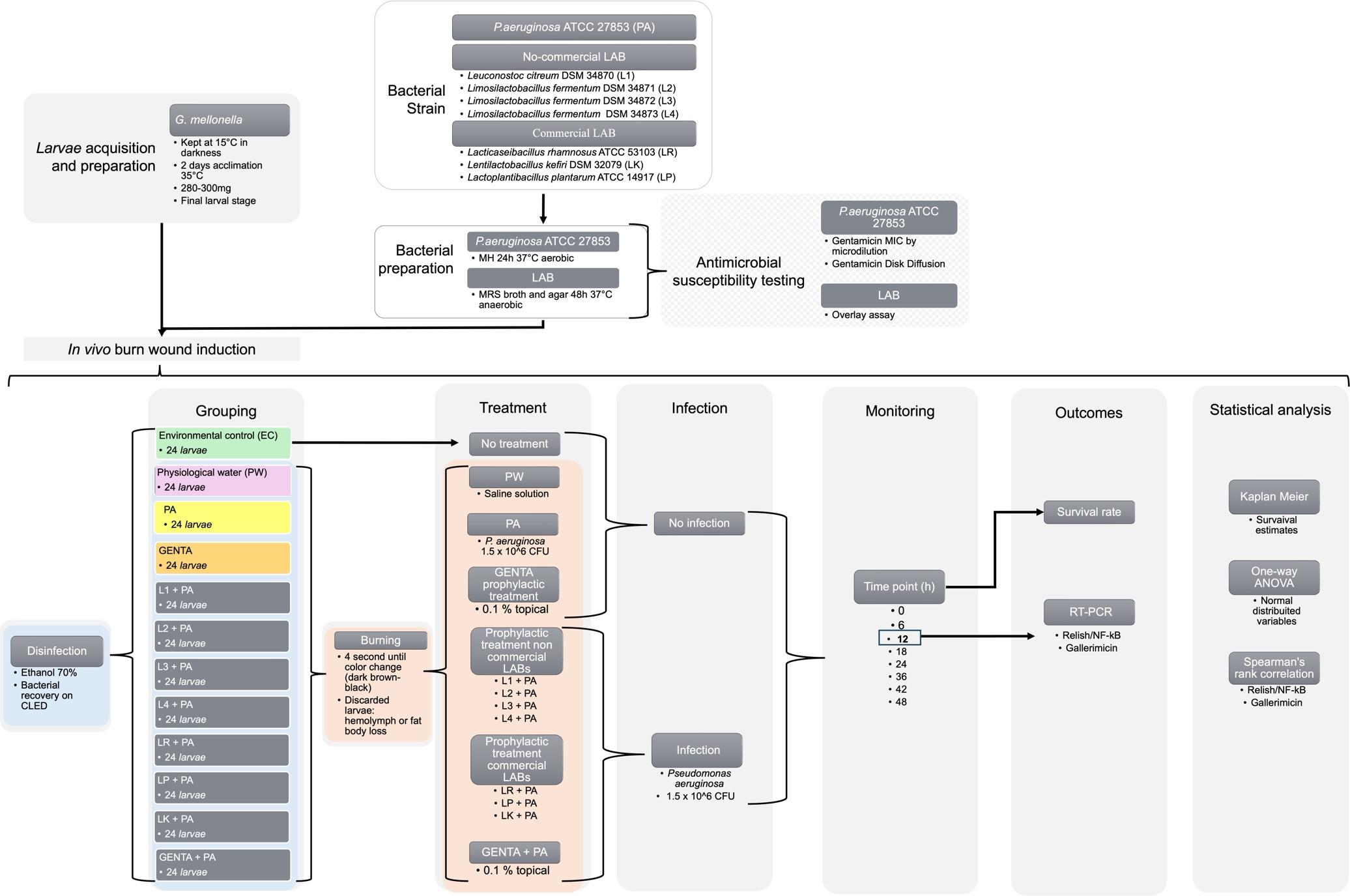

Supplement: Supplementary file 1 [file Table1.docx]
